# Tos4 mediates gene expression homeostasis through interaction with HDAC complexes independently of H3K56 acetylation

**DOI:** 10.1016/j.jbc.2021.100533

**Published:** 2021-03-11

**Authors:** Sophie L. Cooke, Barbara L. Soares, Carolin A. Müller, Conrad A. Nieduszynski, Francisco M. Bastos de Oliveira, Robertus A.M. de Bruin

**Affiliations:** 1 MRC Laboratory Molecular Cell Biology, University College London, London, UK; 2 Instituto de Biofísica Carlos Chagas Filho, Universidade Federal do Rio de Janeiro, Rio de Janeiro, Brazil; 3 Sir William Dunn School of Pathology, University of Oxford, Oxford, UK; 4 Genome Damage and Stability Centre, University of Sussex, Brighton, UK; 5 UCL Cancer Institute, University College London, London, UK

**Keywords:** cell cycle, gene expression, histone deacetylase (HDAC), homeostasis, cell growth, ChIP, chromatin immunoprecipitation, CPT, camptothecin, FACS, fluorescence-activated cell sorted, FHA, forkhead-associated, HAT, histone acetyltransferase, HDAC, histone deacetylase, HU, hydroxyurea, MMS, methyl methanesulfonate, Tos4, **T**arget **o**f **S**wi6-**4**

## Abstract

*Saccharomyces cerevisiae* exhibits gene expression homeostasis, which is defined as the buffering of transcription levels against changes in DNA copy number during the S phase of the cell cycle. It has been suggested that *S. cerevisiae* employs an active mechanism to maintain gene expression homeostasis through Rtt109-Asf1-dependent acetylation of histone H3 on lysine 56 (H3K56). Here, we show that gene expression homeostasis can be achieved independently of H3K56 acetylation by Tos4 (**T**arget **o**f **S**wi6-**4**). Using Nanostring technology, we establish that Tos4-dependent gene expression homeostasis depends on its forkhead-associated (FHA) domain, which is a phosphopeptide recognition domain required to bind histone deacetylases (HDACs). We demonstrate that the mechanism of Tos4-dependent gene expression homeostasis requires its interaction with the Rpd3L HDAC complex. However, this is independent of Rpd3’s well-established roles in both histone deacetylation and controlling the DNA replication timing program, as established by deep sequencing of Fluorescence-Activated Cell Sorted (FACS) S and G2 phase populations. Overall, our data reveals that Tos4 mediates gene expression homeostasis through its FHA domain-dependent interaction with the Rpd3L complex, which is independent of H3K56ac.

Over the course of a cell division cycle, cells must double their cellular content, including their transcriptional output, to maintain cell size homeostasis over generations. It is generally thought that this increase in transcriptional output occurs gradually during the cell cycle, particularly as many cells exhibit a positive correlation between cell size and transcription rate (1, 2). S phase poses a challenge to cells due to the imbalance in gene copy number as a result of the gradual process of DNA replication (Fig. 1*A*), which could cause imbalanced transcription between replicated and unreplicated genes. Multiple bacterial species have been shown to exploit the increased copy number of newly replicated DNA by positioning specific genes close to their replication origin, so their transcript levels will increase upon replication (3). Several recent studies have demonstrated that eukaryotic cells are able to buffer transcription of newly replicated genes during S phase, in both mammalian cells (4, 5, 6) and the budding yeast *Saccharomyces cerevisiae* (7, 8) (Fig. 1*B*). This phenomenon is referred to as gene expression homeostasis, which ensures that transcript levels in S phase are not affected by a gene’s replication. In particular, it has been suggested that *S. cerevisiae* employs an active mechanism to maintain gene expression homeostasis through the histone acetyltransferase (HAT) Rtt109 and its cofactor Asf1 (7), which together acetylate histone H3 on lysine 56 (H3K56) (9).

**Figure 1:**
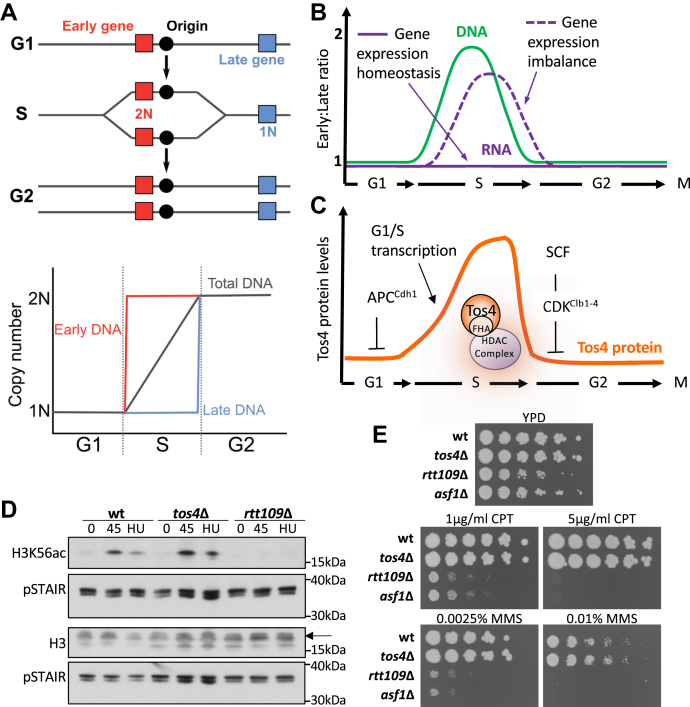
**Tos4 functions independently of Rtt109 and Asf1.***A*, schematic of S phase progression. *Top*, genes that are replicated early will transiently have an increased copy number relative to those replicated late. *Bottom*, thus, while the total DNA content gradually doubles over S phase, individual genes exhibit a step-wise double in copy number. *B*, model of gene expression homeostasis during S phase. All cells exhibit a transient imbalance in DNA copy number between early- and late-replicating genes in S phase (*green line*). Wild-type cells buffer transcription levels against changes in DNA content (gene expression homeostasis, *horizontal purple line*), whereas cells lacking gene expression homeostasis have a transient increase in early-relative to late-replicating genes (gene expression imbalance, *dashed purple line*). *C*, model of the regulation of Tos4 protein levels during the cell cycle. The *TOS4* gene is transcribed as part of the G1/S transcriptional wave. The Tos4 protein is degraded by the APC and SCF complexes outside of S phase. *D*, western blot analysis of H3K56ac levels. Wild-type, *tos4*Δ, and *rtt109*Δ cells were arrested in α-factor (G1), arrested in and released from α-factor for 45 min, representing S phase (S), or arrested in α-factor and released before addition of hydroxyurea (HU), to induce S phase arrest. Western blot analysis was carried out using antibodies against histone H3, H3K56ac, and pSTAIR as a loading control. The *arrow* indicates the relevant band for H3. *E*, sensitivity of strains to the genotoxic agents CPT and MMS. Exponentially growing strains were serially diluted and spotted onto YPD plates containing drugs at the indicated concentrations.

While it is clear that loss of Rtt109, Asf1, or H3K56ac perturbs gene expression homeostasis, these factors are also required for replication-coupled nucleosome assembly, ensuring that histones are appropriately deposited on newly replicated DNA (10). Cells lacking H3K56ac have been shown to exhibit impaired nucleosome assembly on newly replicated genes, and it has been proposed that this may increase transcription, indirectly perturbing gene expression homeostasis (11). This suggests that while loss of acetylation of H3K56 results in loss of gene expression homeostasis, it is not necessarily the molecular mechanism by which gene expression is achieved. In addition, cells lacking Rtt109, Asf1, or H3K56ac exhibit sensitivity to DNA-damaging agents, replication stress, and cell cycle delays (12, 13, 14, 15), but this is more likely a result of impaired replication-coupled nucleosome assembly rather than loss of gene expression homeostasis.

Another regulator of gene expression homeostasis in *S. cerevisiae*, Tos4 (**T**arget **o**f **S**wi6-**4**), was recently identified, but its contribution to gene expression homeostasis has thus far not been explored (7). Tos4 is a nuclear FHA domain-containing protein (16) that is expressed as part of the G1/S transcriptional wave (17). The cell employs multiple mechanisms to restrict the presence of Tos4 protein to S phase, including G1/S transcriptional regulation by both the SBF and MBF transcription factor complexes, and degradation by the APC^Cdh1^ complex and the SCF complex (16, 18, 19) (Fig. 1*C*). The targeted degradation by the SCF complex is mediated by CDK-dependent phosphorylation (18). Interestingly, Tos4 is one of only a limited number of CDK targets that can be phosphorylated by G1 and M phase cyclin-CDK complexes (Cln2, Clb2, and Clb3), but not S phase cyclin (Clb5) CDK complexes (20), further ensuring Tos4’s restriction to only S phase. Additionally, it is significantly upregulated at both the protein and mRNA levels in response to replication stress, which prolongs the time spent in S phase (17). These findings together indicate an important role for Tos4 in S phase. Deleting *TOS4* alongside *DUN1*, which encodes a replication stress checkpoint kinase (21), causes hypersensitivity to hydroxyurea (HU), a replication stress-inducing agent (17). This suggests that Tos4 may have an important role during a prolonged S phase; however, the role of Tos4 has not been established. Interestingly, Tos4 interacts with two histone deacetylase (HDAC) complexes, Rpd3L and Set3c, through its FHA domain (17, 22). A Tos4 FHA mutant exhibits the same synthetic sickness in HU when combined with a *DUN1* deletion (17), suggesting that its role during S phase depends on its interaction with these HDAC complexes. While an initial study suggested that Tos4 functions as a transcription factor (23), there is no evidence to support this. A recent study showed no substantial changes to transcript levels in cells lacking Tos4 (24), and we and others have been unable to isolate chromatin-bound Tos4, either at suggested target sites *via* quantitative PCR ChIP (chromatin immunoprecipitation) or genome-wide *via* ChIP-CHIP analysis (unpublished data and personal communication J. Bahler).

Here we investigate the function and mechanisms of Tos4 in gene expression homeostasis. We show that Tos4 is not required to maintain H3K56ac or for survival in conditions of genotoxic stress, indicating an independent function to Rtt109 and Asf1. We show that Tos4 mediates gene expression homeostasis through its FHA domain, which is required for Tos4’s interaction with the Rpd3L and Set3c histone deacetylase complexes. In addition, we demonstrate a contribution of Rpd3 to gene expression homeostasis, which is independent of its role in histone deacetylation and regulation of DNA replication timing.

## Results

### Tos4 regulates gene expression homeostasis independent of H3K56 acetylation status

Loss of Rtt109, Asf1, or Tos4 results in loss of gene expression homeostasis (7). The HAT Rtt109 and its cofactor Asf1 are responsible for acetylation on H3K56 on newly synthesized histones, which has been proposed as a molecular mechanism for gene expression homeostasis. Tos4 interacts with two HDAC complexes so may also regulate H3K56 acetylation status to maintain gene expression homeostasis. We therefore tested if Tos4 is required for normal H3K56ac dynamics. H3K56 is acetylated on newly synthesized histones in S phase and removed upon S phase completion (14), and deletion of *RTT109* or *ASF1* results in loss of H3K56ac (9). To test H3K56ac levels at different points in the cell cycle, we collected samples from cells arrested in G1 phase with the mating pheromone α-factor, cells released from α-factor for 45 min (representing S phase), and cells arrested in S phase using the replication stress-inducing agent HU. As previously established (14), wild-type cells exhibit increased H3K56ac in S phase and during replication stress compared with a G1 arrest (Fig. 1*D*). No H3K56ac is observed in *rtt109*Δ cells, as expected. However, the *tos4*Δ mutant does not affect H3K56ac, showing very similar levels to those observed in wild-type cells. This shows that Tos4 is not required for proper regulation of H3K56ac and therefore controls gene expression homeostasis in a mechanism independent of H3K56 acetylation status.

Previous studies have shown that Rtt109, Asf1, and H3K56 acetylation are required to maintain genome integrity. Cells lacking these proteins exhibit hypersensitivity to genotoxic agents, such as Camptothecin (CPT) and methyl methanesulfonate (MMS) (15, 25). We therefore tested the sensitivity of the *tos4*Δ mutant to these genotoxic agents. Unlike the *rtt109*Δ and *asf1*Δ mutants, the *tos4*Δ mutant shows similar growth to wild-type cells (Fig. 1*E*). This suggests that the increased sensitivity to genotoxic stress observed upon loss of Rtt109 and Asf1 is unlikely a consequence of impaired gene expression homeostasis. This indicates that studying Tos4 is therefore more likely to be specific to establishing the biological relevance of maintaining gene expression homeostasis.

### Tos4 mediates gene expression homeostasis through its FHA domain

**Figure 2:**
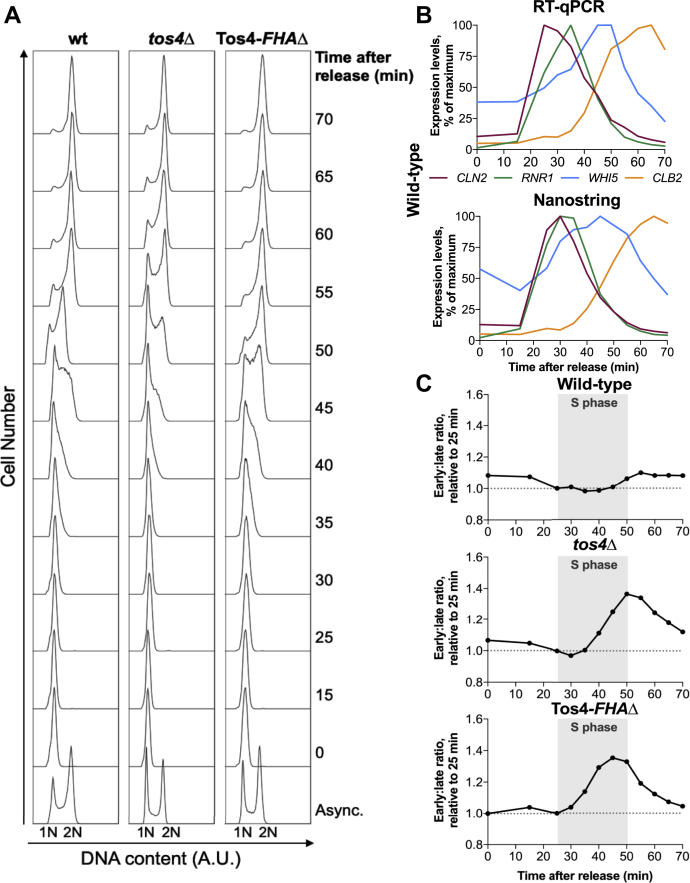
**Tos4 mediates gene expression homeostasis through its FHA domain.** Wild-type, *tos4*Δ, and Tos4-*FHA*Δ cells were arrested in G1 phase using α-factor and released synchronously across the cell cycle. *A*, samples were subject to flow cytometry analysis to confirm cell cycle synchronization. *B*, expression analysis of four cyclic genes by RT-qPCR (*top*) and Nanostring technology (*bottom*) in wild-type cells. *C*, nanostring technology was used to calculate the expression ratio of early-to late-replicating genes. The transcript levels for 14 early-replicating and 13 late-replicating genes were quantified and normalized to levels at 25 min. The average of the early and late genes was calculated before calculating the early:late ratio.

Our work shows that Tos4 does not regulate H3K56ac in *S. cerevisiae*, yet it was previously shown that Tos4 is required for gene expression homeostasis (7). It is therefore likely that Tos4-dependent gene expression homeostasis is mediated through a parallel pathway to Rtt109 and Asf1. In order to study Tos4’s mechanism in gene expression homeostasis, we sought to precisely quantify the mRNA levels of representative early- and late-replicating genes in a synchronous sample. In order to obtain a synchronized cell cycle population, cells were arrested in G1 phase using α-factor and released synchronously. Samples were collected every 5 min in S phase and early G2. In order to check synchronous cell cycle progression, the DNA content was analyzed by flow cytometry (Fig. 2*A*). Additionally, transcript levels of well-characterized periodically transcribed genes (26) were verified by RT-qPCR (Fig. 2*B* and Fig. S1).

We used Nanostring technology to quantify the mRNA levels of early- and late-replicating genes. This technique counts the number of molecules of many specific mRNAs in one sample directly, without relying on reverse transcription or amplification, and is therefore highly quantitative (27). Very early- or late-replicating genes were selected, which have relatively constant transcript levels across the cell cycle, and relatively short transcript half-lives, based on data from multiple studies (28, 29, 30, 31, 32) (Table S2).

Firstly, wild-type samples were subject to analysis by Nanostring technology. The mRNA levels of cyclic genes were also established by RT-qPCR to compare with quantification by Nanostring, and highly similar expression dynamics were observed (Fig. 2*B*). We used Nanostring to quantify the expression levels of 14 early-replicating and 13 late-replicating genes. These values were firstly normalized to 25 min, representing late G1, and subsequently the average of all early- or late-replicating genes was calculated. The average late-replicating gene expression levels were then normalized to the average early-replicating gene levels, to obtain the early:late ratio. As previously reported (7), the early:late ratio remains relatively constant throughout the cell cycle, demonstrating gene expression homeostasis. This ratio does deviate slightly from 1 and actually undergoes a small increase in late S phase, peaking at 1.1 at 55 min. This minor increase is similar to the previously published work (7).

The same analysis was carried out for the *tos4*Δ mutant. Unlike wild-type cells, the *tos4*Δ mutant exhibits a substantial increase in the early:late ratio, which peaks at 1.36 at 50 min, demonstrating a loss of gene expression homeostasis as previously observed (7). Although the previous study used RNA-seq, the authors also observed a similar peak in early:late ratio of approximately 1.4 in mutants lacking gene expression homeostasis (*tos4*Δ, *rtt109*Δ, and *asf1*Δ), suggesting there is good concordance between the two techniques. Published work from our group showed that Tos4’s role in replication stress tolerance, in the absence of the checkpoint transcriptional regulator Dun1, is dependent on its FHA domain, which mediates its interaction with the HDAC complexes Set3c and Rpd3L (17). We therefore tested if loss of gene expression homeostasis is also observed in the Tos4-*FHA*Δ mutant, characterized in our previous study in which just two amino acids (R122 and N161) have been mutated to alanine. The Tos4-*FHA*Δ mutant exhibits a highly similar loss of gene expression homeostasis to the *tos4*Δ mutant, indicating that Tos4’s role in gene expression homeostasis depends upon its interaction with the HDACs.

### Loss of Tos4 does not significantly affect histone acetylation status

**Figure 3:**
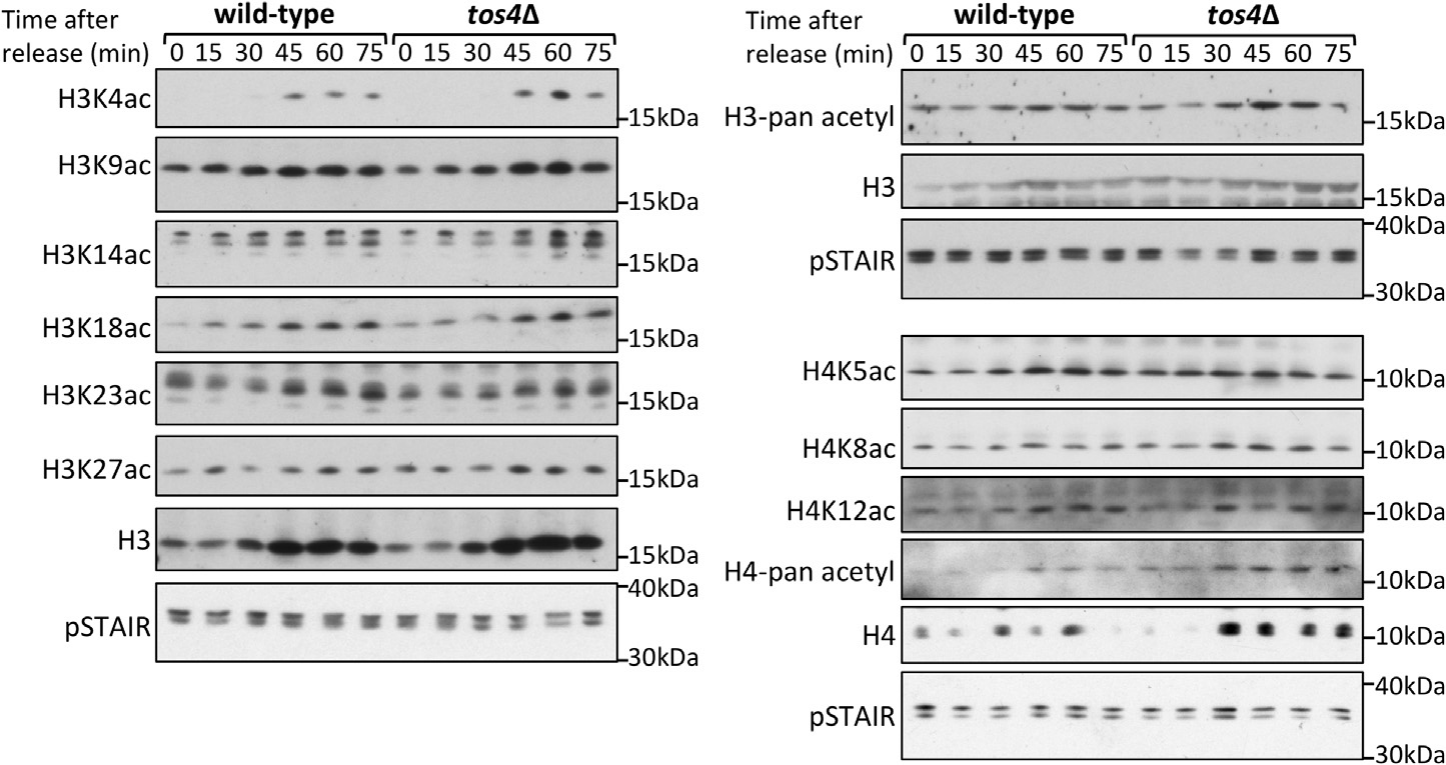
**Cells lacking Tos4 do not have altered histone H3 or H4 acetylation.** Wild-type and *tos4*Δ cells were arrested in and released from G1 phase at 15-min intervals and samples collected for western blot analysis. Antibodies against multiple acetylation marks on histone H3 and H4 were blotted against, including pan-acetyl antibodies that recognize multiple modifications. pSTAIR is included as a loading control.

Our data indicates that while Tos4 does not regulate H3K56ac, its FHA domain, required for its interaction with HDACs, is essential for its role in gene expression homeostasis. This suggests that Tos4 might mediate gene expression homeostasis through regulating the acetylation status at other histone lysine residues. Its interacting HDAC complexes, Rpd3L and Set3c, have been implicated in deacetylation of other lysine residues on histones H3 and H4 (33, 34, 35, 36). We therefore tested if the acetylation status of other lysine residues on H3 or H4 is affected in a *tos4*Δ mutant. We carried out a cell cycle time course after release from α-factor, as we would expect a change in the acetylation status specifically during S phase. We do not observe a significant difference in the acetylation of the lysine residues tested (Fig. 3). This suggests that the mechanism of Tos4-dependent gene expression homeostasis is unlikely through modulation of histone acetylation.

### Tos4 does not regulate replication timing

**Figure 4:**
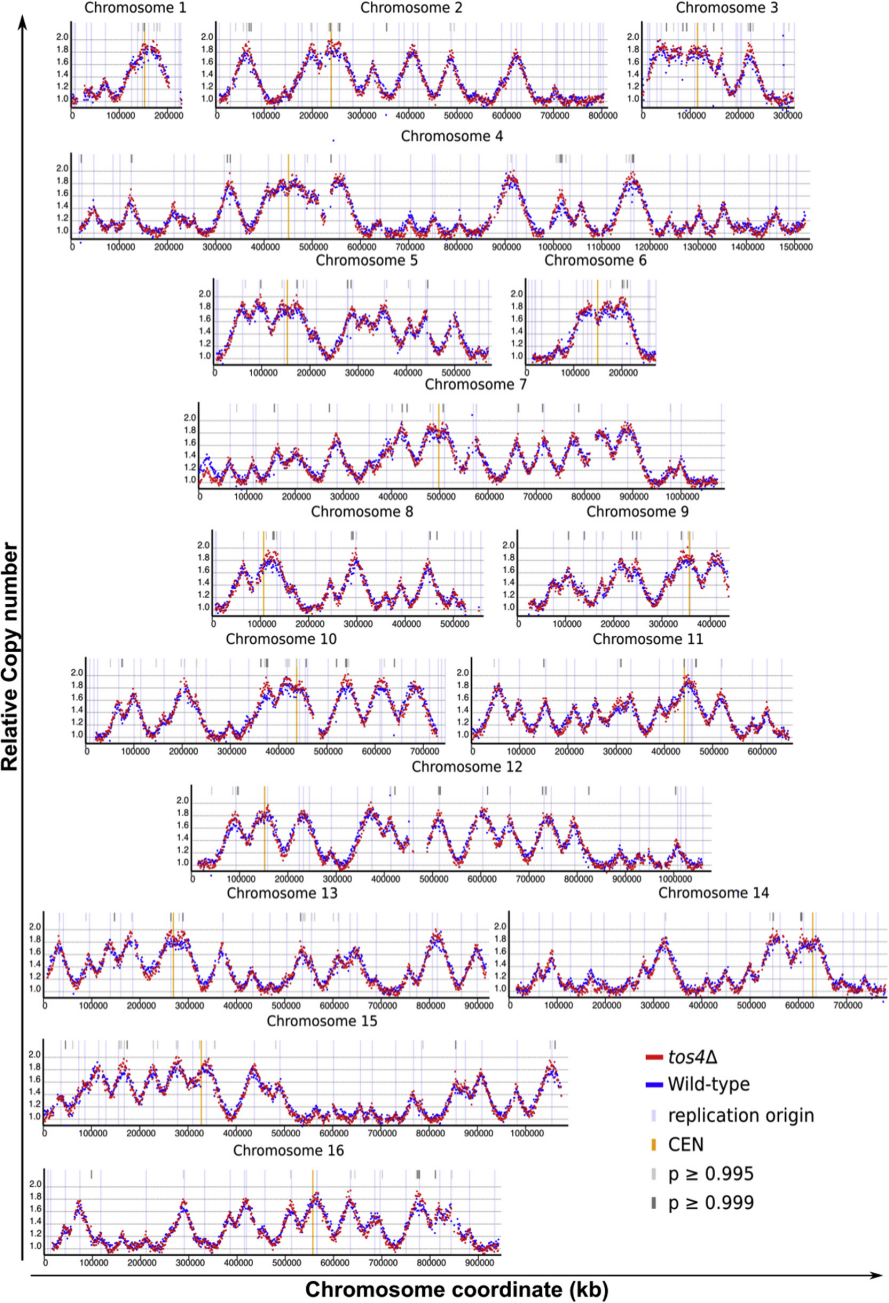
**Global replication timing is unaltered in the *tos4*Δ mutant.** Exponentially growing wild-type and *tos4*Δ cells were subject to sort-seq, in which S and G2 populations were collected by FACS and subject to DNA extraction and deep sequencing. The S phase DNA copy number was calculated relative to the G2 copy number to infer the relative replication timing. Shown are the replication timing plots for the 16 *S. cerevisiae* chromosomes.

We have shown that Tos4’s role in gene expression homeostasis depends upon its FHA domain, which is required for interaction with HDACs. The HDAC Rpd3 has been proposed to have a role in the regulation of the DNA replication timing program (37, 38, 39). Based on this, Tos4, through its regulation of HDAC complexes, could reduce an imbalance in gene expression of early and late-replicating genes by limiting the time window between the firing of early and late origins. If this contributes to the mechanism regulating gene expression homeostasis, then loss of Tos4 would cause early origins to fire earlier and late origins to fire later. To test if Tos4 helps maintain DNA replication timing, we characterized the DNA replication timing program in a *tos4*Δ mutant strain using sort-seq (40). Exponentially growing asynchronous cells were sorted into S phase or G2 phase by FACS. DNA was then extracted from these cells and subjected to deep sequencing. The S phase DNA copy number was compared with the G2 copy number to infer relative replication timing. The *tos4*Δ mutant shows no substantial changes in the timing program compared with wild-type (Fig. 4), demonstrating that Tos4’s mechanism in gene expression homeostasis does not involve regulation of DNA replication timing.

### Rpd3 is required for gene expression homeostasis

**Figure 5:**
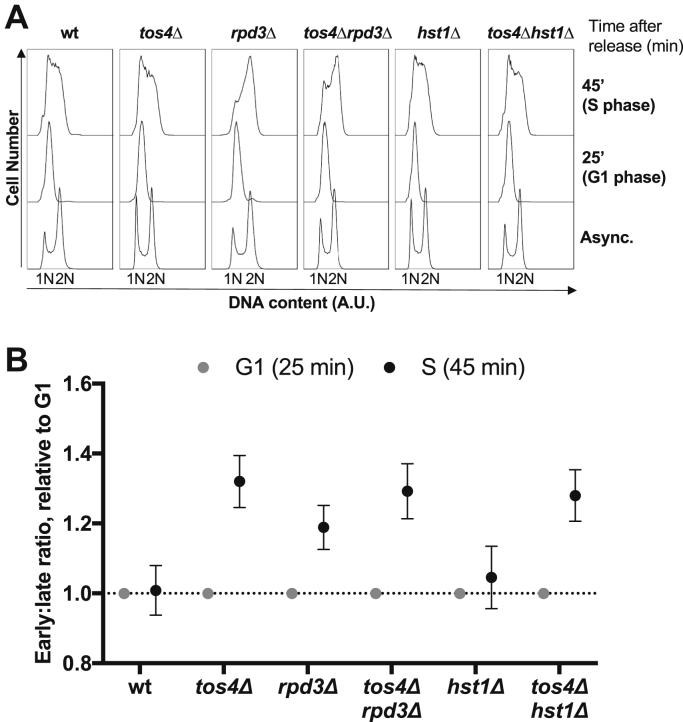
**Rpd3, but not Hst1, is required for gene expression homeostasis.** Wild-type, *tos4*Δ, *rpd3*Δ, *tos4Δrpd3*Δ, *hst1*Δ, and *tos4*Δ*hst1*Δ cells were arrested in G1 phase and released, with samples collected after 25 min (late G1) and 45 min (S phase). *A*, flow cytometry analysis confirms cell cycle synchronization. *B*, nanostring technology was used to calculate the early:late ratio for each strain in late G1 and S phase. As in Figure 2, the expression values for 14 early-replicating and 13 late-replicating genes were calculated. These were normalized to late G1 expression levels, before averaging all early- or late-replicating genes and then the early:late ratio was calculated.

The data presented here indicates that Tos4 regulates gene expression homeostasis through interaction with HDAC complexes *via* its FHA domain. Since Tos4 has been shown by our group and others to interact with the HDACs Rpd3 large complex (Rpd3L) and Set3 complex (17, 22), we investigated the involvement of these HDAC complexes in gene expression homeostasis. If Tos4 regulates gene expression homeostasis *via* these HDAC complexes, we expect HDACΔ mutants to show loss of gene expression homeostasis similarly to *tos4Δ*, moreover a double HDACΔ/*tos4Δ* mutant should not be additive. To test this we generated strains lacking Rpd3 and Hst1, the catalytic subunits of the Rpd3L and Set3 complexes respectively, in both wild-type and *tos4*Δ backgrounds. Cells were arrested in G1 phase using α-factor and samples collected after 25 min release (late G1) and 45 min release (mid S phase) to test if the strains exhibit gene expression homeostasis. Flow cytometry was carried out to confirm synchronization of the cultures (Fig. 5*A*). The cultures show good synchronization, although cells lacking Rpd3 do enter S phase slightly quicker following release from α-factor, as we have previously shown (41). RNA samples were collected at the same time points for analysis by Nanostring (Fig. 5*B*). The data analysis was carried out as described above; for each sample the average value of 14 early-replicating genes normalized to the average of 13 late-replicating genes is presented. All values are normalized to the expression levels in G1 (25 min). As shown previously, there is no increase in the early:late ratio in wild-type cells, while *tos4*Δ cells show an increase from G1 to S phase. Interestingly, *rpd3*Δ cells show a smaller increase in early:late ratio in S phase, suggesting that Rpd3 is required for gene expression homeostasis. The double deletion strain *tos4*Δ*rpd3*Δ shows a similar loss of gene expression homeostasis to the single *rpd3Δ* and *tos4*Δ mutants alone. In contrast, *HST1* deletion does not cause a change in the early:late ratio, suggesting the Set3 complex is not involved in gene expression homeostasis. These data show that Rpd3 has a role in gene expression homeostasis. Importantly it indicates that the mechanism of Tos4-dependent gene expression homeostasis is through its regulation of the Rpd3L complex.

## Discussion

In order to prevent an imbalance in transcription between early- and late-replicating genes during S phase, cells need a mechanism to suppress transcription of replicated genes, in a process termed gene expression homeostasis. Thus far most research into this in *S. cerevisiae* has focused on a potential mechanism for gene expression homeostasis involving H3K56ac, *via* Rtt109 and Asf1. However, as previously suggested, while the loss of H3K56ac correlates with loss of gene expression homeostasis, this could be indirect, due to its effect on deregulated nucleosome assembly in S phase (11). In support of this, recent work has shown that H3K56ac does not direct RNA Polymerase II binding on newly replicated DNA, shedding doubt on its potential function as a transcriptional repressor (42). Here we provide additional evidence for this by establishing an important role for Tos4 in the regulation of gene expression homeostasis though a mechanism independent of H3K56ac. Our data show that the mechanism of Tos4-dependent gene expression homeostasis is likely through its interaction with the Rpd3L complex. Surprisingly, this is unlikely to be mediated through Rpd3’s well-established roles of modulating histone acetylation (34, 35) and controlling the DNA replication timing program (37, 38, 39). Therefore, while the exact mechanism remains to be determined, our data indicates that the molecular mechanism of gene expression homeostasis may involve Tos4-dependent targeted deacetylation of a nonhistone protein likely by the Rpd3L complex.

Although we did not directly compare the loss of Tos4 and Rtt109 upon gene expression homeostasis in this study, previous work showed a highly similar result between the two mutants (7), suggesting that both pathways have a similar contribution to gene expression homeostasis. In addition, the same study reports no increased loss of gene expression homeostasis in a *tos4*Δ *rtt109*Δ double deletion, when compared with either single deletion. While the mechanism by which Tos4/Rpd3 regulates gene expression homeostasis remains a matter of active research, it is possible that it could function through a specific pathway to limit the accessibility of newly replicated regions to transcription machinery. In line with such a mechanism, altering nucleosome occupancy, such as through loss of Rtt109/Asf1, may cause loss of gene expression homeostasis by increasing the accessibility of newly replicated genes to the transcription machinery. Interestingly, in *S. cerevisiae*, histone genes, which are early-replicating, were shown to be able to escape gene expression homeostasis to ensure there are enough histones to package the increasing DNA content in S phase (43). Recent work shows that, while the transcription of histone genes is limited by the copy number, the expression of most genes is limited by the available transcription machinery (44). Tos4/Rpd3 could therefore be involved in limiting the local availability of transcription machinery, that is, the local transcriptional capacity, at replicated genes.

Many roles for Rpd3 have been described; including in control of DNA replication (37, 38, 39), transcriptional regulation (45, 46) and the DNA damage response (47, 48). Further work will therefore be required to understand how Rpd3’s role in gene expression homeostasis relates to its other functions. For example, it is important to consider that Rpd3 is also a member of the Rpd3S complex, which does not interact with Tos4 (22), so additional work will be required to confirm that Rpd3’s function in gene expression homeostasis is mediated specifically *via* the Rpd3L complex.

The phenotypes of cells lacking Rtt109 or Asf1 are severe (12, 13, 14, 15), and we propose that these arise from defects in their functions outside of gene expression homeostasis, such as replication-coupled nucleosome assembly (10). Interestingly, loss of Tos4 does not confer severe fitness defects, which suggests that cells are able to tolerate loss of gene expression homeostasis moderately well. In mammalian cells, it was recently shown that genes encoding subunits of the same protein complex tend to undergo synchronized replication (4), which is likely one mechanism to maintain homeostasis among components of the same protein complex. Additionally, there is a high degree of conservation of replication timing between closely related yeast species (43, 49), and the selection pressure for this may have arisen as a way of mitigating the consequences of a potential loss of gene expression homeostasis.

Our work, along with previous research (17), shows that *tos4*Δ cells do not exhibit severe fitness defects. While loss of Tos4-dependent gene expression homeostasis is well tolerated by the cell in unperturbed conditions, the consequences might be more apparent when cells experience a prolonged S phase due to replication stress. Our work investigating genetic interactions of cells lacking Tos4 in the fission yeast, *Saccharomyces pombe*, in normal conditions as well as during replication stress suggests that loss of Tos4 increases the cell dependence upon components of the gene expression and protein production pathways (unpublished data). In particular, the presence of negative interactors with roles in translation indicates that cells lacking Tos4 may have increased dependence on pathways to maintain proteostasis.

Interestingly, impaired proteostasis has been observed in models of aneuploidy in yeast as well as in higher organisms (50), which is another example of a DNA dosage imbalance. Cells lacking Tos4 instead only have a transient transcription imbalance during S phase, and further work is required to understand whether this is sufficient to cause a notable imbalance in protein dosage in cells lacking gene expression homeostasis. However, while loss of gene expression homeostasis is well tolerated in unperturbed conditions, it is likely to reduce fitness in stress conditions, which in the context of evolutionary pressure suggests an advantage for maintaining gene expression homeostasis. Overall, the work presented here indicates that understanding Tos4’s role will be essential to establish the importance of gene expression homeostasis.

## Experimental procedures

### Yeast growth conditions

The *S. cerevisiae* BY4741 background was used for all experiments, excluding the Nanostring experiments for which the 15Daub (15D) background was used for improved cell cycle synchrony. Strains are listed in Table S1. Strains were derived from wild-type using PCR-based methods (51). All cultures were grown in YPD (Formedium CCM0205) at 30 °C with aeration.

### Cell cycle synchronization

The mating pheromone α-factor (GenScript RP01002) was added to exponentially growing yeast cultures at a final concentration of 0.2 μg/ml. Cultures were incubated for at least 90 min and efficiency of arrest was monitored by number of budding cells. The cultures were then washed with fresh media and released into warm fresh rich media for the rest of the time course. To arrest cells in S phase using HU, cells were arrested in α-factor as described above and released for 15 min before addition of HU (Sigma H8627) at a final concentration of 200 mM and grown in HU for 1 h.

### Analyzing DNA content by flow cytometry

In total, 500 μl of yeast culture was mixed with 1 ml cold 95% ethanol and left at 4 °C for at least 18 h. Fixed cells were pelleted at 5000*g* for 20 min at 4 °C. The pellet was then washed twice in 800 μl of 50 mM sodium citrate buffer pH 7.2, with 10 min incubation at room temperature in between each spin. Then cells were resuspended in 500 μl of 50 mM sodium citrate pH 7.2 containing 20 μg/ml RNase A (Sigma R4875) and 2.5 μM Sytox Green (Thermo Scientific S7020). Cells were incubated for at least 1 h at 37 °C in the dark. Then proteinase K (VWR 390973P) was added at a final concentration of 400 μg/ml and the mixture incubated at 55 °C in the dark for at least an hour. Before analysis by flow cytometry samples were sonicated briefly. The DNA content of 50,000 cells was analyzed using BD LSR II Flow Cytometer. Data was analyzed using FlowJo software.

### Western blots

In total, 10 ml of yeast culture was collected for each condition. Cell pellets were resuspended in 300 μl lysis buffer (50 mM Tris-HCl pH 8, 150 mM NaCl, 7 mM EDTA, 5 mM DTT, cOmplete mini protease inhibitor (Roche 4693124001)). The samples were then vortexed with glass beads (Biospec 11079105) at 4 °C for 20 min. Samples were centrifuged and the supernatant collected for analysis. Samples were loaded onto NuPAGE Novex 4 to 12% Bis-Tris protein gels (Invitrogen, NP0322). Gels were transferred to nitrocellulose membrane by wet transfer. Membranes were incubated with antibodies in PBS-0.2% tween with 5% milk. The following primary antibodies were used at the specified concentrations: H3 (CST 9715, 1:10,000), H3K4ac (Millipore 07-539, 1:1000), H3K9ac (Millipore 07352, 1:5000), H3K14ac (Millipore 07-353, 1:10,000), H3K18ac (Millipore 07-354, 1:5000), H3K23ac (Millipore 07-355, 1:10,000), H3K27ac (Millipore 07-360, 1:15,000), H3K56ac (Millipore 07-677, 1:1000), H3 pan-acetyl (Millipore 06-599, 1:500), H4 (Millipore 05-858, 1:1000), H4K5ac (Millipore 07-327, 1:2000), H4K8ac (Abcam ab15823, 1:1000), H4K12ac (Abcam ab1761, 1:5000), H4 pan-acetyl (Millipore 05-858, 1:1000), and pSTAIR loading control (Sigma-Aldrich P7962, 1:4000). Membranes were developed using ECL films (GE Healthcare Life Sciences, 28906836) and a XOGRAF Compact X4 film processor.

### RNA extraction and RT-qPCR

In total, 20 ml of yeast culture was collected, cells were pelleted and snap frozen at −80 °C. RNA extraction was performed with RNeasy Plus Mini Kit (Qiagen 74134) according to the manufacture’s protocol. Before extraction the pellet was disrupted with glass beads (Biospec 11079105) in RLT buffer supplemented with 1% of β-mercaptoethanol. The RT-qPCR reaction was carried out using One-Step Mesa Green mastermix no ROX (05-SYRT-032XNR), which was supplemented with Euroscript Reverse Transcriptase/RNase inhibitor (Eurogentec RT-0125-ER). Reactions were carried out in a total volume of 14 μl with 80 ng RNA. Reactions were run on a BioRad CFX Connect machine. The data was normalized using *ACT1* as a loading control and analyzed using Ct value method.

### Nanostring

Nanostring elements technology was used. The oligonucleotide probes were supplied in pools by IDT and the reporter and capture tags in pools from Nanostring. All other reagents were supplied by Nanostring. The hybridization reaction proceeded according to the manufacturer’s instructions, using 140 ng total RNA. For the hybridization reaction samples were incubated for 18 h at 67 °C. After hybridization, the hybridized RNA was subject to postreaction cleanup and loaded into a cartridge using the automated Nanostring prep station (configured to high sensitivity). The cartridge was then imaged and analyzed using a nCounter MAX digital analyzer set to count 555 fields of view (maximum sensitivity).

The raw RNA counts were normalized to account for differences in hybridization efficiency between samples. This was performed based on the counts for six positive controls included in each reaction. The geometric mean of the six positive controls was calculated for each reaction, then the geometric means across the 12 reactions were averaged. This averaged value was divided by the geometric mean for each lane to generate the lane-specific normalization factor. The raw counts were then multiplied by this value, thus controlling for experimental variation between samples. For comparison of early- and late-replicating genes, the expression values were firstly normalized to the 25 min timepoint (representing late G1). The average of all early- and late-replicating genes was then calculated, and these were directly normalized to each other to obtain the early:late ratio. The cell cycle genes were normalized to all other housekeeping genes (the early-replicating genes, late-replicating genes, and the mid-replicating gene *ALF1*). They were subsequently normalized to the maximum expression value across the time course. The error bars on Figure 5 were calculated as described previously and represent an approximation of standard error (7). Briefly the variance of early- and late-replicating genes was calculated and divided by the number of genes. These values for early- and late-replicating genes were added together, and the square root of this value was taken.

### Spotting assays

Exponentially growing yeast cultures were centrifuged to concentrate cells to 1 OD_600_/ml. This was subject to five serial fivefold dilutions in H_2_O. The yeasts were spotted onto the appropriate agar plate using a purpose-built pin apparatus.

### DNA replication timing analysis

DNA replication timing analysis was carried out as described elsewhere (40, 49). Briefly, asynchronous wild-type and *tos4*Δ cells were subject to sorting by Fluorescence-Activated Cell Sorting (FACS) using DNA content to collect an S phase and G2 phase population. DNA from these samples was subject to deep sequencing. The average copy number of genomic regions in the S phase population was normalized to the G2 population to infer DNA replication timing. Statistical analysis was carried out through calculation of the z-score genome-wide, to identify 1 kb bins with a significant difference between strains, as described previously (52).

## Data availability

All the data described in the article are contained within the article.

## Supporting information

This article contains supporting information.

## Conflict of interest

The authors declare no competing interests.
