# Supplementary material for: Tos4 mediates gene expression homeostasis through interaction with HDAC complexes independently of H3K56 acetylation
Source: J Biol Chem. 2021 Mar 11;296:100533. doi: 10.1016/j.jbc.2021.100533 (PMC8054192; doi:10.1016/j.jbc.2021.100533)
Supplement: Supplemental Figure S1 and Tables S1–S2 [file mmc1.pdf]

Supporting Information: Tos4 mediates gene expression homeostasis through interaction with HDAC complexes independently of H3K56 acetylation.

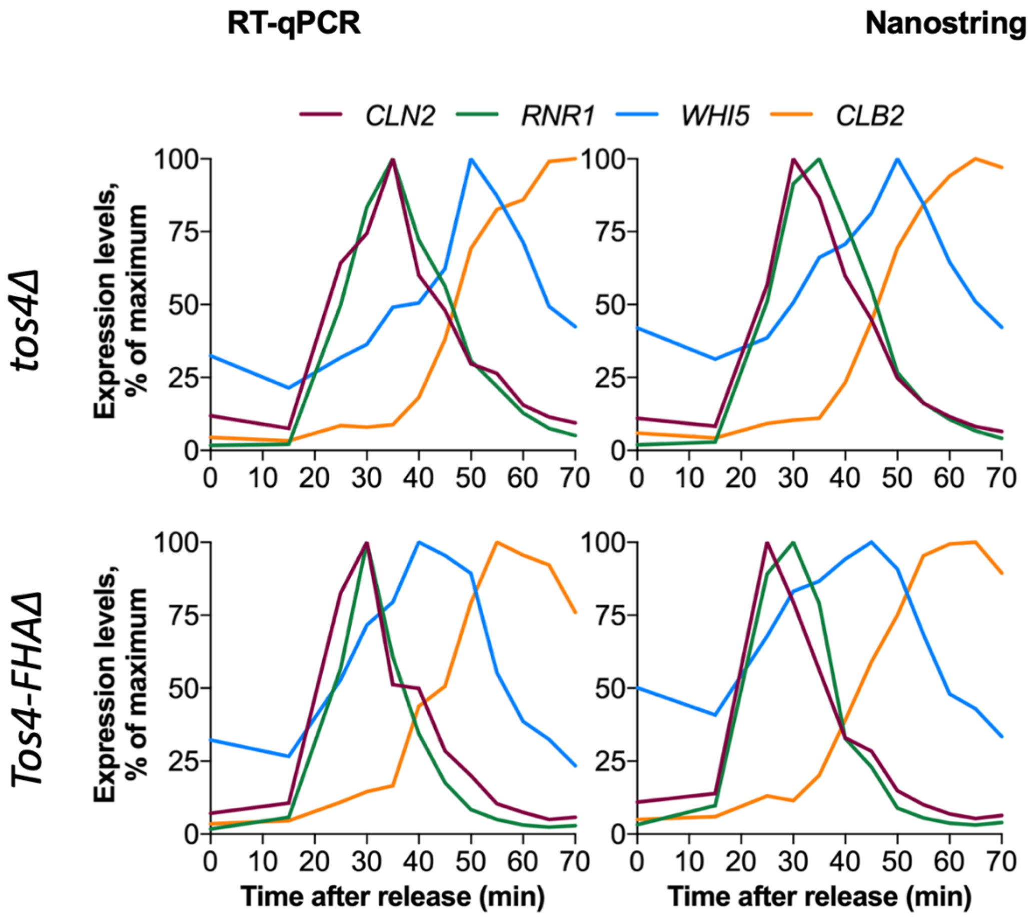

Supplementary Figure 1: Expression of periodic genes for the tos4Δ and Tos4-FHAΔ time-courses (related to Figure 2). Expression of four cell cycle-regulated genes was tested by RT-qPCR (left) and Nanostring (right). Transcript levels are presented relative to the maximum expression level over the time-course for that gene.

Strain	Genotype
RBY1	15D wild-type, *MATa, ade1, leu2-3, 112 his2, trp1-1, ura3Δns, bar1Δ*
RBY280	RBY1 + *rpd3::URA3*
RBY325	RBY1 + *tos4::TRP1*
RBY752	RBY1 + *Tos4-(R122A;N161A) -3HA::Kan* (Tos4-*FHAΔ*)
RBY768	RBY325 + *rpd3::URA3*
RBY774	RBY1 + *hst1::KanMX6*
RBY776	RBY325 + *hst1::KanMX6*
RBY686	BY4741 wild-type, *his3Δ1 leu2Δ0 met15Δ0 ura3Δ0 bar1Δ::HIS3*
RBY684	RBY686 + *rtt109::KanMX6*
RBY685	RBY686 + *asf1::KanMX6*
RBY687	RBY686 + *tos4::Ura3*

Supplementary Table 1: *S. cerevisiae* strains used in this study

Early-replicating genes	Late-replicating genes
BNA5	*ALT2*
CLU1	*CCT6*
GAL3	*FYV6*
HEM6	*LEU9*
HIT1	*MDM30*
PEX31	*PAC1*
POF1	*SEC63*
RPC11	*SNU23*
RSM26	*SRB2*
STB6	*TUM1*
TRM12	*UBP8*
YCL002C	*YHK8*
YJR056C	*YOR1*
YPP1	

Supplementary Table 2: Genes analysed by Nanostring.
